# Zinc Supplementation Initiated Prior to or During Pregnancy Modestly Impacted Maternal Status and High Prevalence of Hypozincemia in Pregnancy and Lactation: The Women First Preconception Maternal Nutrition Trial

**DOI:** 10.1016/j.tjnut.2024.04.018

**Published:** 2024-04-16

**Authors:** Jennifer F Kemp, K Michael Hambidge, Jamie L Westcott, Sumera Aziz Ali, Sarah Saleem, Ana Garcés, Lester Figueroa, Manjunath S Somannavar, Shivaprasad S Goudar, Julie M Long, Audrey E Hendricks, Nancy F Krebs, the Women First Preconception Maternal Nutrition Trial Group, Sangappa M Dhaded, Sunil S Vernekar, Veena R Herekar, S Yogeshkumar, Elizabeth M McClure, Abhik Das, Vanessa R Thorsten, Richard J Derman, Robert L Goldenberg, Marion W Koso-Thomas

**Affiliations:** 7 KLE Academy of Higher Education and Research’s Jawaharlal Nehru Medical College, Belagavi, India; 8 RTI International, North Carolina, United States; 9 Thomas Jefferson University, Philadelphia, PA, United States; 10 Columbia University, New York, NY, United States; 11 Pregnancy and Perinatology Branch, NICHD/NIH, Rockville, MD, United States; 1 Department of Pediatrics, Section of Nutrition, University of Colorado School of Medicine, Aurora, CO, United States; 2 Department of Community Health Sciences, Aga Khan University, Karachi, Pakistan; 3 Maternal Infant Health Center, Instituto de Nutrición de Centro América y Panamá (INCAP), Guatemala City, Guatemala; 4 Women’s and Children’s Health Research Unit, KLE Academy of Higher Education & Research’s JN Medical College, Belagavi, Karnataka, India; 5 Department of Biomedical Informatics, University of Colorado School of Medicine, Aurora, CO, United States; 6 Department of Mathematical and Statistical Sciences, University of Colorado Denver, Denver, CO, United States

**Keywords:** zinc deficiency, small-quantity lipid-based nutrient supplement (SQ-LNS), inflammation, birth length, birth weight, pregnancy, lactation

## Abstract

**Background:**

Data regarding effects of small-quantity-lipid-based nutrient supplements (SQ-LNS) on maternal serum zinc concentrations (SZC) in pregnancy and lactation are limited.

**Objectives:**

The objectives of this study were to evaluate the effect of preconception compared with prenatal zinc supplementation (compared with control) on maternal SZC and hypozincemia during pregnancy and early lactation in women in low-resource settings, and assess associations with birth anthropometry.

**Methods:**

From ∼100 women/arm at each of 3 sites (Guatemala, India, and Pakistan) of the Women First Preconception Maternal Nutrition trial, we compared SZC at 12- and 34-wk gestation (*n* = 651 and 838, respectively) and 3-mo postpartum (*n* = 742) in women randomly assigned to daily SQ-LNS containing 15 mg zinc from ≥3 mo before conception (preconception, arm 1), from ∼12 wk gestation through delivery (early pregnancy, arm 2) or not at all (control, arm 3). Birth anthropometry was examined for newborns with ultrasound-determined gestational age. Statistical analyses were performed separately for each time point.

**Results:**

At 12-wk gestation and 3-mo postpartum, no statistical differences in mean SZC were observed among arms. At 34-wk, mean SZC for arms 1 and 2 were significantly higher than for arm 3 (50.3, 50.8, 47.8 μg/dL, respectively; *P* = 0.005). Results were not impacted by correction for inflammation or albumin concentrations. Prevalence of hypozincemia at 12-wk (<56 μg/dL) was 23% in Guatemala, 26% in India, and 65% in Pakistan; at 34 wk (<50 μg/dL), 36% in Guatemala, 48% in India, and 74% in Pakistan; and at 3-mo postpartum (<66 μg/dL) 79% in Guatemala, 91% in India, and 92% in Pakistan. Maternal hypozincemia at 34-wk was associated with lower birth length-for-age *Z*-scores (all sites *P* = 0.013, Pakistan *P* = 0.008) and weight-for-age *Z-*scores (all sites *P* = 0.017, Pakistan *P* = 0.022).

**Conclusions:**

Despite daily zinc supplementation for ≥7 mo, high rates of maternal hypozincemia were observed. The association of hypozincemia with impaired fetal growth suggests widespread zinc deficiency in these settings.

This trial is registered at clinicaltrials.gov as #NCT01883193.

## Introduction

The basic and ubiquitous molecular biology of zinc provides compelling evidence for its essentiality for growth and development of the embryo and fetus and for optimal function of the placenta [1]. Abundant data from animal models document the deleterious effects of severe zinc deficiency at virtually all stages of gestation, including congenital malformations, fetal death, and abnormal parturition [[2], [3], [4]]. In human pregnancy, adverse outcomes attributable to zinc deficiency have been harder to document, even in populations with considerable risk of moderate dietary deficiency. Indeed, the capacity for homeostatic adjustments to increase intestinal absorption of dietary zinc in late pregnancy [5,6] may partially mitigate the effects of marginal intake. The challenge of assessing zinc status, and thus determining zinc deficiency, further complicates linking seemingly insufficient dietary intake with adverse outcomes [2]. This challenge is accentuated during pregnancy when a physiologic decline in circulating zinc concentrations is well documented and is not fully attributable to hemodilution [7,8]. Although lower cutoffs for serum zinc concentration (SZC) for healthy people, including during pregnancy and lactation, have been proposed, functional outcomes have not been linked to them [2,8]. From systematic reviews of zinc supplementation trials, which used a wide range of zinc doses, the only adverse outcome responsive to maternal zinc supplementation that has withstood scrutiny in most [9,10] but not all [11] reviews is the incidence of preterm birth.

A compelling gap in the zinc story is a paucity of data on the effects of zinc supplementation commenced prior to conception on longitudinal maternal zinc status and fetal outcomes. In this report, we compare the impact of daily zinc supplementation (as zinc sulfate), administered as a constituent of a small-quantity lipid-based nutrient supplement (SQ-LNS) formulated for pregnancy [12], that was initiated prior to conception, at the end of the first trimester, or not at all, in the multicountry Women First (WF) Preconception Maternal Nutrition trial [13]. To our knowledge, these are the first data to be reported on the impact of SQ-LNS on these outcomes. The trial also allowed comparison of the intervention’s impact among 3 settings with vastly different cultures and dietary intakes [14] and a range of socio-economic status, although all generally low.

The principal objective of this analysis was to determine the effect of the SQ-LNS intervention on maternal SZC and the frequency of hypozincemia in early and late pregnancy and at 3 mo postpartum, according to intervention arm. Second, we examined whether zinc status was associated with newborn anthropometry and with preterm birth.

## Methods

### Study design

This was a secondary analysis of data collected from the WF trial [13,15]. Briefly, the WF trial was an individually randomized controlled trial investigating timing of maternal SQ-LNS (Nutriset) on fetal growth. Nonpregnant women were enrolled and randomly assigned to 1 of 3 arms. Supplementation was initiated ≥3 mo prior to conception (arm 1); at ∼12 wk gestation (arm 2); or not at all (control arm 3) in women in Guatemala, India, Pakistan, and the Democratic Republic of the Congo (DRC), all participating sites of the Global Network for Women’s and Children’s Health Research [16]. At the time the SQ-LNS was initiated (at preconception in arm 1 and at the end of the first trimester in arm 2), women who were underweight (BMI <20 kg/m^2^) or found to have inadequate gestational weight gain were also provided a balanced protein-energy supplement (Nutriset) that provided ≤300 kcal and 11 g of protein (∼15% of energy), without additional supplemental micronutrients. Participants were recruited from 53 geographic catchment areas or clusters. Due to limitations of cold chain capacity in the DRC, data presented here represent only the other 3 sites. The daily LNS provided 15 mg zinc; other specific nutrient compositions of the SQ-LNS have been presented previously [13]. This amount of zinc is slightly greater than the Recommended Dietary Allowance (RDA) [17]; it is consistent with amounts in the UNIMMAP multiple micronutrient supplement for pregnancy developed by the WHO [12,18] and was considered to offer favorable acceptability for long-term consumption, especially for women randomly assigned to the preconception group in the WF trial.

Assessment of zinc status was based on measurement of zinc concentrations in serum samples of nonfasting women obtained at ∼12- and 34-wk gestation and 3-mo postpartum. All materials and supplies used during collection, storage, and analyses of the samples were zinc-free and provided to the research sites by the University of Colorado Denver (UCD) team. On-site training specific to avoiding environmental zinc contamination during sample collection and processing was delivered by the UCD team. SZC results were augmented with ancillary laboratory assays for albumin and biomarkers of systemic inflammation.

### Subjects

Blood samples were collected at 12-wk gestation from women in arms 1 and 2, and at 34-wk gestation and 3 mo postpartum from participants in all arms, excluding women in arm 3 from India due to absence of ethical approval for collections from these participants. The 12-wk blood sample was collected from women in arm 2 prior to commencement of the daily study supplement, and thus, they served as the control group at that time. The samples were obtained from the first 100 participants per arm per site who successfully entered the pregnancy phase of the study and who provided written consent to provide biospecimens. The participants included in the analyses of birth anthropometry had a first-trimester ultrasound to determine gestational age.

Details of procedures for newborn anthropometry (length, weight, and head circumference) have been reported previously [15]. Measurements were converted to gestational age-adjusted length-, weight-, head circumference- and weight-to-length ratio-for-age *Z*-scores (LAZ, WAZ, HCAZ, and WLRAZ, respectively) using INTERGROWTH-21st fetal growth charts [19].

### Ethical considerations

The study was registered at clinicaltrials.gov as NCT01883193 and was approved by the Colorado Multiple Institutional Review Board, the local and/or national ethics committees for each of the 3 participating sites (registered with the United States Office of Human Research Protection and with Federal-wide Assurance in place) and the data coordinating center. Written informed consent was obtained from all participants. The consent form was translated into the local languages at each site and presented to the participants by native speakers (Spanish for Guatemala, Kannada for Belagavi, and Sindhi for Pakistan). Throughout the intervention and follow-up phases of the trial, a data monitoring committee designated by the *Eunice Kennedy Shriver* National Institute of Child Health and Human Development monitored data quality and safety of the trial. The study protocol [12] is provided online at https://www.ncbi.nlm.nih.gov/pmc/articles/PMC4000057.

### Sample collection and laboratory procedures

Specimens were collected with zinc-free materials provided to each site by the University of Colorado research group, including powder- and zinc-free nitrile gloves (#C9905PF, Showa Clean-Dex), Safety-Multifly needles, and serum S-Monovette blood collection tubes (Sarstedt). The blood was allowed to clot for 30 min before centrifugation and transfer of serum to cryovials. All materials used for the collection, transfer, and storage of biospecimens were confirmed to be zinc-free prior to use in this study. Serum was stored in −80°C freezers located at each site until shipment on dry ice to the Pediatric Nutrition Laboratory at the University of Colorado Anschutz Medical Campus (Aurora, CO). Upon receipt, all samples were stored at −80°C until analyzed.

SZC was measured in the Pediatric Nutrition Laboratory by inductively coupled plasma mass spectrometry (ICP-MS, Agilent Technologies 7700x) following the method used by Forrer et al. [20]. Calibration standards were prepared with Supelco TraceCert Inductively Coupled Plasma (ICP) zinc standard solution and internal standard solutions with TraceCert ICP yttrium standard solution (#75594 and #75592, respectively, Aldrich Chemical Co, Inc.). The ICP-MS was optimized to achieve the highest counts at zinc mass 66 with 40 ng/mL natural zinc standard and the lowest counts at mass 66 with 2% (vol:vol) nitric acid (HNO_3_) (#A467, OPTIMA, Fisher Scientific). The serum and zinc standards were diluted 300 times using ∼3 parts per billion yttrium solution in 2% HNO_3_. A standard curve (0.70 μg/mL, 1.00 μg/mL, and 1.25 μg/mL) was established; SZC was determined against the standard curve. SeroNorm Trace Elements serum level 1 (#SR201413, Accurate Chemical, and Scientific Corporation) and an inhouse plasma pool were analyzed after every 5 serum samples, alternating between the 2, within each analytical run. Within assay precision of internal controls was <4%, and between assay precision was 12.3%. The limit of detection for this method is 0.1 μg zinc/dL.

C-reactive protein (CRP) was measured at the Pediatric Nutrition Laboratory in serum samples from the Guatemalan and Pakistani sites using a sandwich immunoassay with electrochemiluminescent detection (#K151STD, Meso Scale Discovery). The serum samples collected in India were analyzed by immunoturbidimetry (Siemens Dimension) at the KLE Hospital Clinical Lab (Belagavi, Karnataka, India). Alpha-1-acid glycoprotein (AGP) was measured using a sandwich enzyme-linked immunoassay (#DAGP00, R&D Systems, Bio-Techne Corp); samples from Guatemala and Pakistan were measured at the Pediatric Nutrition Laboratory, whereas those collected in India were assayed at the Basic Science Research Center in Belagavi, India. Albumin concentrations were measured by the Colorado Clinical Translational Research Center core laboratory (Aurora, CO) using an enzymatic assay (Beckman Coulter). The same serum pool and SeroNorm controls were measured regularly at all analytical locations to monitor consistency between the analysis methods conducted in the United States and India.

To minimize potential batch effects, samples from Guatemala and Pakistan were balanced across runs by randomizing within site, arm, and longitudinal time points. Samples from India were not randomized but instead ran chronologically, starting with 12-wk and then 34-wk time points. The 3-mo postpartum samples for India were included in the randomization with Guatemala and Pakistan and were analyzed in the Pediatric Nutrition Laboratory. The study design randomly assigned individual participant IDs across arms, which also minimized batch effects with arm. Samples with results below the lower limits of quantitation for each biomarker were repeated and subsequently removed from the data set if still below this level.

### Statistical methods

STATA software v17 [21] was used to complete descriptive statistics and tests, along with the albumin correction for zinc concentrations. Statistical Software R v4.2.1 was used for all other statistical analyses. Logistic regression for binary outcomes and linear regression for continuous outcomes were employed, using the glm function in the stat R package with either the family=binomial or default, respectively. To compare adjusted probabilities or means by category (e.g., arm, site), we used the emmeans function in the emmeans R package (v1.8.9) [22]. For the biomarkers measured, chi-squared tests of independence were used to detect whether the number of participants in each arm differed by batch.

#### Descriptive statistics

Differences in baseline characteristics between intervention arms were evaluated using unpaired t tests with unequal variance (12-wk) and 1-way ANOVA with Tukey post-hoc testing (34-wk and 3-mo) for continuous variables. Categorical variables were compared using the Fisher’s exact test. An α level of <0.05 was considered statistically significant. CRP and AGP concentrations were considered elevated if >5 mg/L and >1 g/L, respectively [23,24].

#### Assessment of SZC and hypozincemia by arm and site

Linear models with the glm function in base R were used to investigate the relationship between continuous zinc concentrations, arm, and site separately for 12-wk gestation, 34-wk gestation, and 3-mo postpartum time points. Logistic regression, using the emmeans R package (v1.8.9) [22], was used to investigate the relationship between hypozincemia and site while adjusting for arm, and for arm while adjusting for site. Hypozincemia was defined as <56 μg/dL (1st trimester), <50 μg/dL (2nd/3rd trimester), and <66 μg/dL at 3-mo postpartum [8]. Association between arm and zinc (either mean or hypozincemia) was assessed by combining all sites while controlling for site as a covariate in the model as well as stratified by site.

#### Correction of SZC for hypoalbuminemia

As developed by Jørgensen et al. [25], a simple linear regression model with albumin-corrected SZC was applied to adjust zinc concentrations with albumin concentrations. The model was adjusted for arm and stratified by site and time point. The highest value of the lowest decile (LD_alb_) for albumin was calculated for each group. The following equation was used for the albumin correction: Zinc_corrected_ = Zinc_measured_ − ($\hat{\beta }$) ∗(Albumin_measured_ − LD_Alb_)) [25].

#### Association between birth anthropometry and hypozincemia

A multiple linear regression model was used to assess the relationship between hypozincemia and continuous newborn anthropometry measures, adjusting for gestational age using the INTERGROWTH-21^st^ fetal growth charts [19]. Arm was included as a covariate to adjust for study design, and analyses were completed across all sites (including site as a covariate) and stratified by site.

#### Association between mean SZC and preterm birth (<37 wk)

Logistic regression was used to assess the relationship between mean zinc concentrations and preterm birth as defined by gestational age <37 wk [26]. Zinc concentration was modeled as the primary predictor with prematurity as the outcome, adjusting for arm and site, as well as stratified by site.

#### Sensitivity to inflammation

For all models described above, sensitivity of the SZC results to inflammation was assessed by including CRP and AGP concentrations as covariates in the model.

#### Outliers and model robustness

Outliers for zinc, CRP, and AGP were identified within each arm, timepoint, and site, and were defined as observations 3 interquartile units above or below the 3rd and 1st quartiles, respectively. To evaluate model robustness for exclusion of outliers and adjusting for study site clusters, secondary analyses were performed adjusting for cluster or excluding outliers.

#### Multiple testing

Nominal statistical significance was assessed at a *P* value of 0.05. Statistical significance adjusting for multiple testing within 12-wk, 34-wk, and 3-mo models was assessed using the Bonferroni correction at 0.05/14 = 0.004 where the 14 primary models include association of 5 birth outcomes (LAZ, WAZ, HCAZ, WLRAZ, and preterm birth) plus arm and site by both continuous SZC and categorical hypozincemia. Post-hoc analyses of categorical predictors (i.e., site and arm) using a Tukey adjustment for multiple testing were performed using the *emmeans* function from the *emmeans* R package v1.4.4 [22].

## Results

Data were collected between December 2013 and March 2017. One hundred sixteen outliers were identified with 5 SZC only, 7 AGP only, 98 CRP only, and 6 outliers in both AGP and CRP. Outcomes were consistent if outliers were removed or if adjusted for cluster (birth outcomes only); results reported thus do not remove outliers or adjust for cluster.

The baseline characteristics of the participants in this study, their birth outcomes, the prevalence of systemic inflammation, and mean serum albumin concentrations are shown in Table 1. For each site and arm, ∼35% of the participating women who became pregnant had biochemical measurements at the 3 time points (Supplementary Figure 1 Consort Diagram). No statistically significant differences in baseline characteristics were observed among women in different arms at 12-wk (all *P-*unadj > 0.05), whereas at 34-wk and 3-mo postpartum, more women in control arm 3 had no or only primary education and higher parity. Similar to the primary study [15], newborns of women who provided blood samples for biochemical measurements had lower birth LAZ in the control arm 3 compared with intervention at the preconception arm 1 or early pregnancy arm 2 (Table 1).

**TABLE 1:** Maternal characteristics at enrollment, maternal biomarkers and infant birth outcomes by intervention arm^1^ and time point for combined sites.^2^

Variable	12-wk Gestation^3^		34-wk Gestation^4^			3-mo Postpartum^4^		
	Arm 1 PreconceptionSupplement	Arm 2 BeforeSupplementInitiation	Arm 1 PreconceptionSupplement	Arm 2 PrenatalSupplement	Arm 3 Control	Arm 1 PreconceptionSupplement	Arm 2 PrenatalSupplement	Arm 3 Control
Women with serum zinc, n^5^								
Combined sites	322	329	315	307	216	277	271	194
Guatemala	105	113	107	112	108	82	93	91
India^6^	112	110	99	89	—	95	81	—
Pakistan	105	106	109	106	108	100	97	103
Maternal age								
Age, y	23.3 ± 4.1	23.3 ± 3.9	23.3 ± 4.1	23.6 ± 3.9	24.3 ± 4.3	23.3 ± 4.1	23.7 ± 3.9	24.5 ± 4.4
<20 y	61 (19)	57 (17)	60 (19)	46 (15)	36 (17)	53 (19)	41 (15)	30 (15)
≥20 y	261 (81)	272 (83)	255 (81)	262 (85)	181 (83)	224 (81)	230 (85)	164 (85)
Body mass index								
BMI, kg/m^2^	21.7 ± 4.4	21.8 ± 4.4	21.7 ± 4.4	21.9 ± 4.5	22.3 ± 4.6	21.4 ± 4.3	21.7 ± 4.4	22.1 ± 4.7
<18.5	88 (27)	80 (24)	85 (27)	73 (24)	42 (19)	79 (29)	68 (21)	43 (22)
18.5–24.9	167 (52)	179 (54)	163 (52)	165 (54)	121 (56)	147 (53)	146 (54)	107 (55)
≥25	67 (21)	70 (21)	67 (21)	69 (22)	53 (25)	51 (18)	57 (21)	44 (23)
Maternal education^7^								
No formal schooling	103 (32)	97 (29)	105 (33)	94 (31)	103 (47)	94 (34)	85 (31)	97 (50)
Primary	83 (26)	108 (33)	86 (27)	102 (33)	89 (41)	69 (25)	84 (31)	75 (39)
Secondary	136 (42)	124 (38)	124 (39)	112 (36)	25 (12)	114 (41)	102 (38)	22 (11)
Parity^8^								
0 (nulliparous)	86 (27)	64 (19)	83 (26)	52 (17)	34 (16)	74 (27)	44 (16)	32 (16)
1	100 (31)	116 (35)	100 (32)	109 (36)	61 (28)	86 (31)	93 (34)	52 (27)
≥2	136 (42)	149 (45)	132 (42)	146 (48)	121 (56)	117 (42)	134 (49)	110 (57)
SES^9^								
None (0–present)	3 (0.9)	1 (0.3)	4 (1.3)	1 (0.3)	4 (1.9)	3 (1.1)	0 (0)	4 (2.1)
1–2 present	75 (23)	60 (18)	74 (23)	61 (20)	52 (24)	64 (23)	57 (21)	47 (24)
3-4 present	167 (52)	178 (54)	164 (52)	161 (52)	114 (53)	144 (52)	139 (51)	101 (52)
5-6 present	77 (24)	90 (27)	73 (23)	84 (27)	46 (21)	66 (24)	75 (28)	42 (22)
Maternal biomarkers								
CRP (%>5 mg/L)	116 (36)	120 (37)	124 (39)	128 (42)	94 (44)	60 (22)	51 (19)	32 (16)
AGP (%>1 g/L)	88 (27)	106 (32)	19 (6)	21 (7)	13 (6)	65 (23)	50 (19)	30 (15)
Albumin, g/dL	4.1 ± 0.05	4.1 ± 0.6	3.4 ± 0.3	3.4 ± 0.3	3.4 ± 0.3	4.6 ± 0.3	4.6 ± 0.3	4.6 ± 0.3
Preterm birth (<37 wk gestation)								
Combined sites	31 (11)	27 (10)	27 (10)	20 (7)	20 (12)	--	--	--
Guatemala	4 (5)	6 (7)	4 (5)	6 (7)	6 (7)	--	--	--
India^4^	9 (10)	10 (11)	5 (6)	5 (6)	--	--	--	--
Pakistan	18 (19)	11 (11)	18 (18)	9 (10)	14 (16)	--	--	--
Infant birth anthropometry (*z*-scores adjusted for gestational age)								
N	262	267	267	262	162	--	--	--
Length, cm^10^	47.7 ± 2.1	47.8 ± 2.3	47.6 ± 2.1	47.9 ± 2.1	47.3 ± 2.4	--	--	--
LAZ^11^	-0.68 ± 0.96	-0.61 ± 1.01	-0.71 ± 0.97	-0.60 ± 0.99	-0.86 ± 1.11	--	--	--
Weight, g	2804 ± 392	2818 ± 464	2793 ± 395	2840 ± 424	2812 ± 416	--	--	--
WAZ	-0.91 ± 0.87	-0.86 ± 0.97	-0.93 ± 0.89	-0.85 ± 0.94	-0.86 ± 0.92	--	--	--
HC, cm	33.1 ± 1.3	33.1 ± 1.5	33.1 ± 1.3	33.2 ± 1.4	33.0 ± 1.5	--	--	--
HCAZ	-0.42 ± 1.03	-0.43 ± 1.07	-0.45 ± 1.02	-0.42 ± 1.05	-0.46 ± 1.11	--	--	--
WLRAZ	-1.26 ± 1.26	-1.16 ± 1.33	-1.28 ± 1.27	-1.17 ± 1.33	-1.06 ± 1.24	--	--	--

Abbreviations: AGP, Alpha-1-acid glycoprotein; CRP, C-reactive protein; HC, head circumference; HCA*Z*, head circumference-for-age *Z*-score; LA*Z*, length-for-age *Z*-score; SES, socio-economic status; WA*Z*, weight-for-age *Z*-score; WLRA*Z*, weight-for-length ratio-for-age *Z*-score.

1 Arm 1 commenced the supplement ≥3 mo prior to conception and continued through delivery; arm 2 commenced the same intervention late in the first trimester (after sample collection) and continued until delivery; arm 3 (Control) received no study supplements.

2 Results presented as n, n (%), or mean ± SD.

3 Baseline maternal characteristics and infant outcomes are not statistically different between arms at 12-wk (1-way ANOVA and Fisher’s exact test *P* > 0.05).

4 Unless otherwise indicated by different superscripts within a row and timepoint and described in the footnotes below, no significant differences were found among arms at 34-wk gestation or at 3-mo postpartum.

5 Sample sizes vary over time due to not obtaining blood from some participants at each time point and additional participants being included in any one group to obtain the sample size goal.

6 Control arm (Arm 3) not available for India at 34-wk or 3-mo postpartum.

7 Maternal education is different among arms at 34-wk (Fisher’s exact test *P* < 0.0001) and 3-mo postpartum (Fisher’s exact test *P* < 0.0001).

8 Parity is different among arms at 34-wk (Fisher’s exact test *P* = 0.002) and 3-mo postpartum (Fisher’s exact test *P* = 0.003).

9 The socio-economic status (SES) tally provides the number of indicators available from the following list: electricity, improved water source, sanitation, man-made flooring, improved cooking fuels, and household assets.

10 Birth length is lower in Arm 3 than in Arm 2 for participants in the 34-wk gestation (Tukey's *P* = 0.020, 95% CI −0.07, −1.08).

11 Length-for-age *Z*-score is lower in Arm 3 than in Arm 2 at 34-wk gestation (Tukey's *P* = 0.035, 95% CI −0.014, −0.49).

### SZC and hypozincemia by arm and site

**FIGURE 1:**
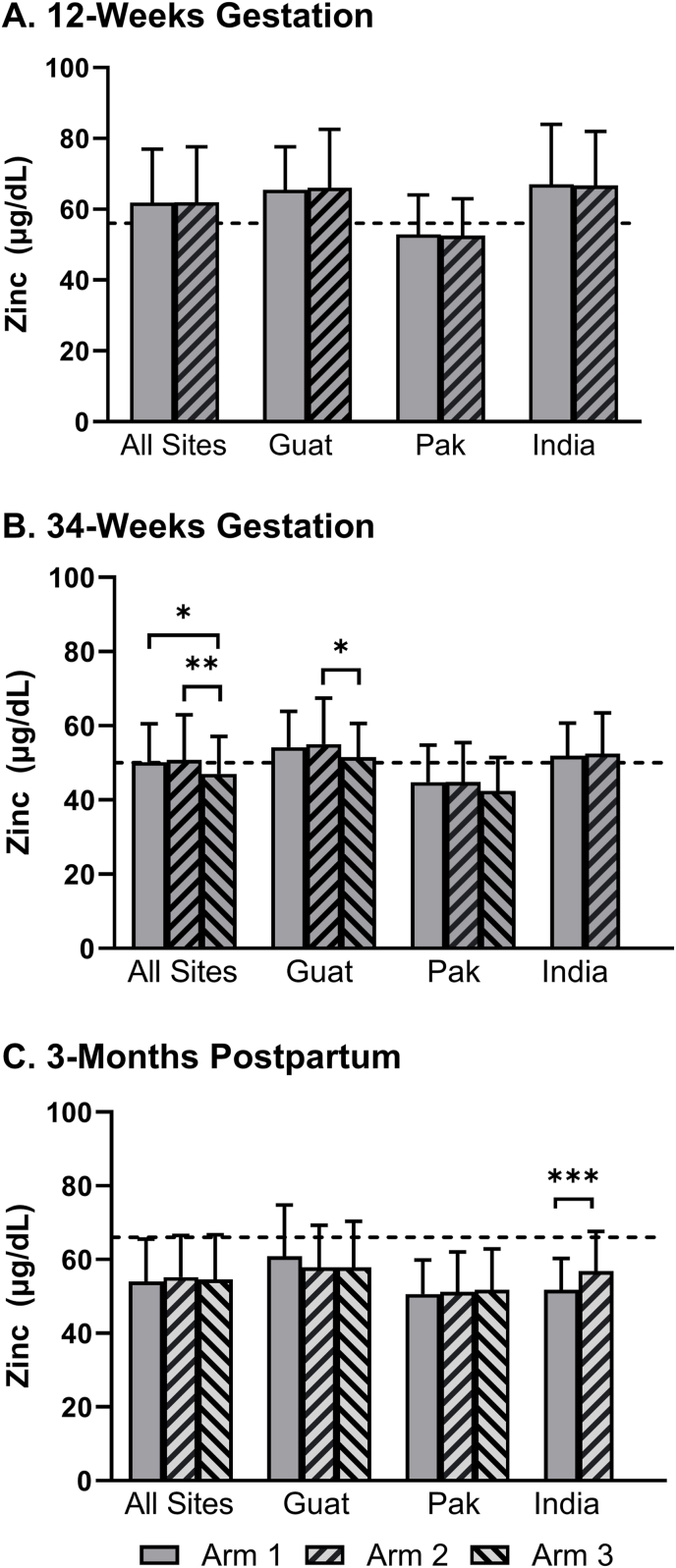
Serum zinc concentration (μg/dL) by site and arm. Mean (±SD) serum zinc concentration by site and arm at (A) 12-wk gestation, (B) 34-wk gestation, and (C) 3-mo postpartum. Arm difference *P* values denoted by ∗(< 0.05), ∗∗(< 0.005), and ∗∗∗(< 0.0005). Dotted line marks deficiency cutoff at each time point [8]. Arm 1 initiated the supplement ≥3 mo prior to conception and continued through delivery; arm 2 commenced the same intervention late in the first trimester (after sample collection) and continued until delivery; arm 3 (control) received no study supplements. Guat, Guatemala; Pak, Pakistan.

The effect of the intervention on mean SZC was evaluated for combined and individual sites at 12- and 34-wk gestation and 3-mo postpartum (Table 2). No statistical differences in means were observed between arms 1 and 2 (preconception and early pregnancy, respectively) at 12-wk, either for combined sites or for any individual sites. At 34-wk, although SZC did not differ significantly between arms 1 and arm 2, means for both intervention arms were significantly higher compared with controls (arm 3) (*P* = 0.023 and 0.005, respectively). This result was driven primarily by results from Guatemala, but a trend of similar magnitude was observed in Pakistan. Similar comparisons were not available for India due to the absence of biospecimen collection in the control arm 3. At 3-mo postpartum, mean SZC were not statistically different by arm for combined sites or separately for Guatemala or Pakistan. In India, at 3-mo postpartum, the mean SZC was higher in arm 2 than in arm 1 (*P* < 0.001) (Figure 1). Results were consistent when including CRP and AGP as covariates to adjust for inflammation. Additionally, when the mean SZC was corrected for hypoalbuminemia, the SZC values declined slightly in all groups and time points except for India at 12-wk gestation.

**TABLE 2:** Serum zinc concentration (μg/dL) by arm at 12- and 34-wk gestation and 3-mo postpartum for combined and individual sites.

Site	Arm^1^	N	Mean (95% CI)	Difference between Means			Global *P* value^3^
				Arm Comparison	Difference (95% CI)	Pairwise *P* value^2^	
12-wk gestation							
Combined sites	1	322	61.8 (60.3, 63.3)	1–2	0.02 (−2.14, 2.17)	0.99	-
	2	329	61.8 (60.3, 63.3)				
Guatemala	1	105	65.5 (62.7, 68.3)	1–2	−0.56 (−4.44, 3.32)	0.78	-
	2	113	66.0 (63.3, 68.7)				
India	1	112	67.0 (64.0, 70.0)	1–2	0.29 (−3.96, 4.53)	0.90	-
	2	110	66.7 (63.7, 69.8)				
Pakistan	1	105	52.9 (50.8, 55.0)	1–2	0.33 (−2.57, 3.23)	0.82	-
	2	106	52.6 (50.5, 54.6)				
34-wk gestation							
Combined sites	1	315	50.3 (49.2, 51.4)	1–2	−0.48 (−2.07, 1.11)	0.83	0.005
	2	307	50.8 (49.7, 51.9)	1–3	2.47 (0.63, 4.30)	0.023	
	3	216	47.8 (46.4, 49.3)	2–3	2.94 (1.11, 4.77)	0.005	
Guatemala	1	107	54.2 (52.2, 56.2)	1–2	−0.77 (−3.56, 2.02)	0.85	0.040
	2	112	55.0 (53.1, 57.0)	1–3	2.68 (−0.14, 5.49)	0.15	
	3	108	51.6 (49.6, 53.6)	2–3	3.45 (0.67, 6.22)	0.041	
India	1	99	51.9 (50.0, 53.9)	1–2	−0.53 (−3.37, 2.30)	0.71	0.71
	2	89	52.5 (50.4, 54.5)				
Pakistan	1	109	44.8 (42.9, 46.6)	1–2	−0.12 (−2.75, 2.52)	1.00	0.13
	2	106	44.9 (43.0, 46.8)	1–3	2.28 (-0.34, 4.90)	0.20	
	3	108	42.5 (40.6, 44.4)	2–3	2.40 (-0.24, 5.04)	0.18	
3-mo postpartum							
Combined sites	1	277	54.3 (53.0, 55.6)	1–2	-0.95 (-2.81, 0.92)	0.58	0.60
	2	271	55.2 (53.9, 56.6)	1–3	-0.25 (-2.41, 1.90)	0.97	
	3	194	54.6 (52.8, 56.3)	2–3	0.69 (-1.45, 2.83)	0.80	
Guatemala	1	82	60.8 (58.1, 63.6)	1–2	2.94 (-0.80, 6.68)	0.27	0.21
	2	93	57.9 (55.3, 60.5)	1–3	3.00 (-0.76, 6.76)	0.26	
	3	91	57.8 (55.2, 60.4)	2–3	0.06 (-3.58, 3.70)	1.00	
India	1	95	51.8 (49.8, 53.7)	1–2	-5.16 (-8.00, -2.31)	0.0004	0.0004
	2	81	56.9 (54.8, 59.0)				
Pakistan	1	100	50.7 (48.6, 52.7)	1–2	-0.59 (-3.49, 2.32)	0.92	0.74
	2	97	51.2 (49.2, 53.3)	1–3	-1.13 (-3.99, 1.73)	0.72	
	3	103	51.8 (49.8, 53.8)	2–3	-0.54 (-3.43, 2.34)	0.93	

^2,3^ Multiple linear regression was used with continuous zinc as the outcome and arm as the primary predictor. All results were untransformed to enable interpretability in standard units. Combined site analysis is adjusted for site. Tukey’s *post-hoc* adjustment was used for pairwise comparisons.

1 Arm 1 commenced the supplement ≥3 mo prior to conception and continued through delivery; arm 2 commenced the same intervention late in the first trimester (after sample collection) and continued until delivery; arm 3 (Control) received no study supplements. No arm 3 data was available in the Indian site.

**FIGURE 2:**
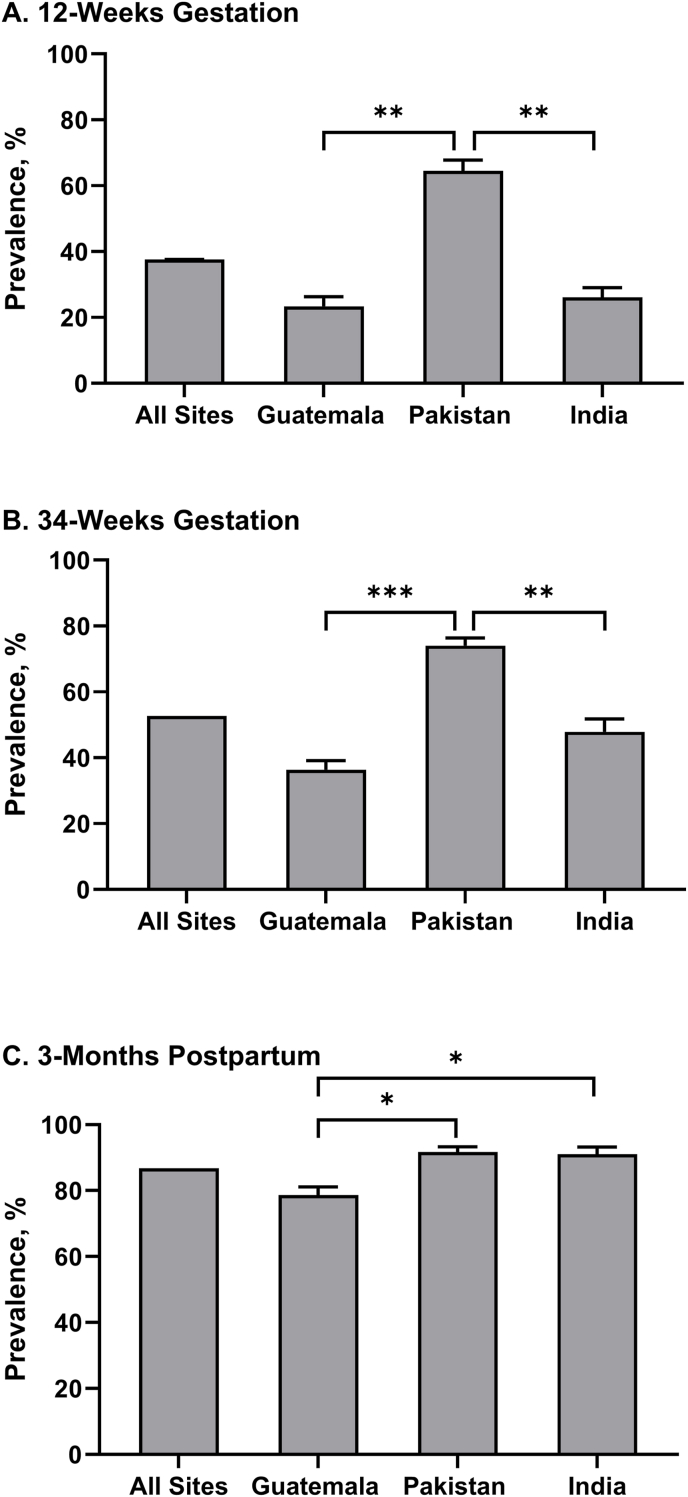
Prevalence of hypozincemia by site. Prevalence, % (±SE) of hypozincemia by site and time point; (A) 12-wk gestation, (B) 34-wk gestation, and (C) 3-mo postpartum. Hypozincemia was defined as <56 μg/dL (1st trimester), <50 μg/dL (2nd/3rd trimester), and <66 μg/dL at 3-mo postpartum [8]. Site difference *P* values denoted by ∗(< 0.05), ∗∗(< 0.005), and ∗∗∗(< 0.0005).

No significant association between hypozincemia and treatment arm was observed for combined sites or within any site. The prevalence of hypozincemia, adjusting for stage of gestation [8], differed by site after adjusting for treatment arm (Figure 2). At all time points, Pakistan had significantly greater prevalence of hypozincemia (64.5%, 74.0%, and 91.7% at 12-wk, 34-wk, and 3-mo, respectively) compared with Guatemala (23.4%, 36.4%, and 78.6%). Pakistan also had significantly greater prevalence of hypozincemia compared with India except at 3-mo postpartum (India prevalence 26.1%, 47.9%, and 91.0% at 12-wk, 34-wk, and 3-mo, respectively).

### Association between hypozincemia and birth size and preterm birth

Women experiencing hypozincemia at 34-wk had, on average, infants with lower LAZ and WAZ at birth than women not experiencing hypozincemia after adjusting for intervention arm (Table 3). These results were nominally significant (i.e., not adjusting for multiple comparisons) and were primarily driven by Pakistan, although the direction of effect (i.e., lower *Z*-scores for mothers with hypozincemia) was seen in Guatemala and India, as well. No significant associations of birth outcomes with hypozincemia at 12-wk gestation were observed. Finally, no significant associations were observed between SZC at any point or intervention arm and premature birth. Results were robust to adjustment for CRP and AGP, albumin, cluster, and exclusion of outliers.

**TABLE 3:** Association of birth anthropometric outcomes with hypozincemia adjusting for treatment arm.^1^

Birth outcome	Site	Time point	N	Hypozincemia Adjusted Mean *Z*-score (95% CI)	No Hypozincemia Adjusted Mean *Z*-score (95% CI)	*P* value
LA*Z*	Combined Sites	12-wk	529	−0.61 (−0.75, −0.46)	−0.66 (−0.77, −0.56)	0.55
		34-wk	691	−0.81 (−0.92, −0.70)	−0.61 (−0.73, −0.49)	0.013^2^
	Guatemala	12-wk	175	−0.58 (−0.86, −0.29)	−0.74 (−0.89, −0.59)	0.31
		34-wk	255	−0.79 (−0.97, −0.61)	−0.73 (−0.86, −0.59)	0.60
	India	12-wk	169	−0.42 (−0.72, −0.11)	−0.66 (−0.82, −0.49)	0.18
		34-wk	165	−0.69 (−0.91, −0.46)	−0.56 (−0.76, −0.35)	0.40
	Pakistan	12-wk	185	−0.67 (−0.87, −0.48)	−0.53 (−0.79, −0.27)	0.39
		34-wk	271	−0.83 (−0.99, −0.67)	−0.40 (−0.67, −0.13)	0.008^2^
WA*Z*	Combined Sites	12-wk	529	−0.83 (−0.97, −0.69)	−0.92 (−1.02, −0.82)	0.32
		34-wk	691	−1.01 (−1.11, −0.91)	−0.84 (−0.94, −0.73)	0.017^3^
	Guatemala	12-wk	175	−0.46 (−0.72, −0.20)	−0.70 (−0.84, −0.56)	0.12
		34-wk	255	−0.72 (−0.88, −0.55)	−0.67 (−0.80, −0.55)	0.66
	India	12-wk	169	−1.00 (−1.28, −0.73)	−1.16 (−1.32, −1.01)	0.32
		34-wk	165	−1.25 (−1.45, −1.05)	−1.08 (−1.26, −0.89)	0.20
	Pakistan	12-wk	185	−0.91 (−1.09, −0.73)	−0.85 (−1.09, −0.61)	0.69
		34-wk	271	−0.99 (−1.12, −0.85)	−0.67 (−0.90, −0.44)	0.022^3^
HCA*Z*	Combined Sites	12-wk	528	−0.36 (−0.52, −0.20)	−0.46 (−0.58, −0.35)	0.32
		34-wk	690	−0.53 (−0.64, −0.41)	−0.40 (−0.52, −0.28)	0.13
	Guatemala	12-wk	175	−0.07 (−0.40, 0.26)	−0.25 (−0.42, −0.07)	0.35
		34-wk	255	−0.34 (−0.55, −0.13)	−0.18 (−0.34, −0.03)	0.23
	India	12-wk	169	−0.31 (−0.63, 0.01)	−0.60 (−0.78, -0.43)	0.12
		34-wk	165	−0.61 (−0.85, −0.38)	−0.54 (−0.75, −0.32)	0.63
	Pakistan	12-wk	184	−0.56 (−0.76, −0.37)	−0.47 (−0.73, −0.21)	0.59
		34-wk	270	−0.59 (−0.74, −0.44)	−0.45 (−0.71, −0.19)	0.35
WLRA*Z*	Combined Sites	12-wk	525	−1.17 (−1.35, −0.98)	−1.25 (−1.39, −1.11)	0.52
		34-wk	685	−1.33 (−1.47, −1.19)	−1.18 (−1.32, −1.03)	0.13
	Guatemala	12-wk	175	−0.53 (−0.92, −0.14)	−0.87 (−1.07, −0.66)	0.13
		34-wk	255	−0.89 (−1.13, −0.64)	−0.84 (−1.02, −0.66)	0.77
	India	12-wk	168	−1.54 (−1.92, −1.16)	−1.71 (−1.92, −1.50)	0.44
		34-wk	164	−1.82 (−2.10, −1.55)	−1.62 (−1.87, −1.37)	0.29
	Pakistan	12-wk	182	−1.25 (−1.49, −1.00)	−1.08 (−1.41, −0.75)	0.42
		34-wk	266	−1.24 (−1.43, −1.05)	−1.00 (−1.32, −0.69)	0.21

Abbreviations: HCAZ, head circumference-for-age Z-score; LAZ, length-for-age Z-score; WAZ, weight-for-age Z-score; WLRAZ, weight-for-length ratio-for-age Z-score.

1 *Z*-scores calculated from INTERGROWTH-21^st^ fetal growth charts [19].

2 Length-for-gestational age *Z-*score (LA*Z*) at birth is nominally statistically different between hypozincemic and not hypozincemic women in Pakistan and combined sites at 34-wk gestation.

3 Weight-for-gestational age *Z*-score (WA*Z*) at birth is nominally statistically different between hypozincemic and not hypozincemic women in Pakistan and combined sites at 34-wk gestation.

## Discussion

We examined zinc status and the frequency of hypozincemia in pregnant and lactating women living in diverse low-resource settings who had received supplemental zinc through multiple-micronutrient-fortified SQ-LNS. The supplement was initiated either prior to conception (arm 1), at the end of the first trimester (arm 2) (or not at all, arm 3), and continued through pregnancy. The principal finding of this analysis is that initiation of the supplement ≥3 mo prior to conception did not result in higher mean SZC at the end of the first trimester compared with women who had had no supplementation, a finding that was consistent for all 3 sites. In contrast, at 34-wk gestation, maternal SZC were higher for both intervention arms compared with controls. With the supplementation discontinued at delivery, at 3-mo postpartum, SZC did not differ among intervention arms. Using previously published cutoffs for pregnancy and lactation [8], rates of hypozincemia exceeded 20% in all sites at all time points and were >60% in Pakistan; rates exceeded ∼80% for all sites at 3-mo postpartum. Nominally significant associations were observed between 3rd trimester zinc status and birth length and weight, whereas no association was observed with preterm delivery.

Three outcomes of this analysis warrant highlighting. First, the association of hypozincemia at 34-wk with lower birth size contrasts with systematic reviews, which have generally found reduced rates of preterm delivery associated with zinc supplementation but without detectable benefit on fetal growth [[9], [10], [11]]. Although hypozincemia was not associated with preterm delivery in this study, as a secondary outcome, the sample size and rates of prematurity were likely too small to detect such a relationship.

Second, maternal SZC at 34-wk gestation were significantly higher for the preconception and pregnancy groups (arms 1 and 2, respectively) compared with the control arm 3. Since this result was observed at 34-wk and not in early gestation, it may be related to the quantitatively greater demands of the conceptus at a time of rapid fetal growth. Even with enhanced zinc absorption in the 3rd trimester [5,6,27], however, these results convincingly support the premise that such adaptation is insufficient to compensate for chronic marginally low-dietary zinc intakes (with moderately high phytate) observed in the participants [14]. Although the results for the Guatemala site were the main drivers of this serum zinc response, a similar trend was observed for Pakistan (similar comparisons not available for India).

Third, using proposed stage of pregnancy and postpartum adjusted cutoffs [8], a substantial prevalence of hypozincemia was evident throughout the study despite women in the preconception group receiving the zinc supplement (as a constituent of SQ-LNS) daily for ≥6 mo and an average of ∼12 mo prior to the 12-wk biospecimen collections [15]. Rates of hypozincemia exceeded 60% in Pakistan in both early and late pregnancy and were >20% for the other 2 sites at 12-wk, and rates approached 50% by 34-wk. Our observations are consistent with a dose-response meta-analysis that found only a modest association of zinc intake with SZC during pregnancy [28]. Correction for systemic inflammation and serum albumin did not provide an apparent explanation for the hypozincemia, as these biomarkers were typically within normal limits. Moreover, the impact of inflammation on SZC in adults is unclear [[29], [30], [31]]. High-dose iron supplementation in pregnancy has been associated with lower SZC [7], but the SQ-LNS used in WF contained only 20 mg iron, a dose less than the RDA for pregnancy [17].

The rates of hypozincemia observed in late pregnancy in the WF participants in India and Pakistan (48% and 74%, respectively) are similar to or higher than indicated by limited data in the literature [[32], [33], [34]]. The quantity of supplemental zinc (15 mg) used in the WF study was half that of SQ-LNS preparations used in other pregnancy trials [12,35]. Combined with estimated dietary zinc intakes of the WF participants, however, total daily intake for the last two-thirds of pregnancy would be predicted to be comfortably above estimated average requirement of 9.5 mg/d [14,17]. At 3-mo postpartum, when the SQ-LNS supplement had been discontinued, and typical zinc output in milk for fully lactating women is ∼1 mg/d [36,37], 80%–90% of the participants were hypozincemic. Data to establish serum cutoffs for early lactation are particularly limited, and these results for WF participants raise the question of the suitability of the 66 μg/dL cutoff used in this analysis [8]. However, data from a randomized zinc supplementation trial conducted in healthy, fully lactating women in Colorado indicated that means for both placebo and zinc supplement (15 mg/d) groups exceeded 80 μg/dL by 3-mo postpartum [36]. Overall, the high rates of hypozincemia observed in the participants through pregnancy and early lactation; the association of hypozincemia with birth anthropometry; and the epidemiologic evidence of widespread zinc deficiency in women of reproductive age in similar low- and middle-income settings [32,33,38,39], support a conclusion that the risk of zinc deficiency was high in the WF participants. In these contexts, the amount of zinc provided may have been insufficient.

A strength of this study was the inclusion of a treatment arm that commenced the trial intervention several months prior to conception. The inclusion of preplanned serum zinc assays in early and late gestation and postpartum provided unique information on the effects of the zinc dose used in the Women First trial, which was low for SQ-LNS but similar to other prenatal multiple micronutrient supplements, e.g., UNIMMAP [40]. The standardized collection procedures and analyses primarily by a single, experienced trace element laboratory were additional strengths. Inclusion of multiple sites allowed useful comparisons of the effects of the zinc supplements in different cultures with very different diets [14] and a range of socio-economic status. This study also had limitations. A baseline preconception SZC would likely have been informative for interpretation of the other results presented, but this was logistically impractical due to the study design of enrolling many women who did not become pregnant. Bioavailability data on the zinc in the SQ-LNS would have potentially been informative. Finally, more comprehensive characterization of the WF participants’ zinc status would have been achieved with inclusion of biospecimens from the study site in the DRC and from the control arm for the site in India. Nevertheless, these data add substantially to the evidence for zinc status of pregnant and lactating women in diverse low-resource settings.

In summary, this analysis examined the effects of zinc supplementation within a daily SQ-LNS preparation consumed prior to and throughout pregnancy and identified detectable effects on maternal zinc status, which was associated with improved fetal growth. The study was not sufficiently powered to test an association with preterm delivery. A somewhat surprising and not fully explained finding was the persistence of a high or, for the participants in Pakistan, a very high prevalence of hypozincemia across gestation and early lactation, despite good compliance (based on sachet counts and maternal report) with the supplement for many months [15]. The findings suggest that the prevalence of zinc deficiency was high in these participants, and optimal intake of zinc over the course of a reproductive cycle in similar low-resource settings is yet to be determined.

## Author contributions

The authors’ responsibilities were as follows – NFK, KMH: designed the research study; KMH, NFK, JLW, SS, AG, SSG, RJD, RLG, MKT: provided oversight of the field research performed by SAA, LF, MSS, SMD, SSV, and VRH; JFK, JML, SY, MSS: collected data in the field and/or performed laboratory analyses; AEH, JFK: conducted the statistical analyses using input from EMM, AD, and VRT; NFK, KMH, AEH, JFK: drafted the paper; and all authors: read and approved the final manuscript.

## Conflict of interest

The authors declare no conflicts of interest.

## Funding

This study was supported by the Bill & Melinda Gates Foundation (OPP1055867), the National Institutes of Health *Eunice Kennedy Shriver* National Institute of Child Health and Human Development and the Office of Dietary Supplements (U10 HD 076474 and UG1 HD 076474). Sponsors had no involvement or restrictions regarding publication.

## Data Availability

Data described in the manuscript, code book and analytic code will be made available upon request to the corresponding author and pending approval. Data from the primary and follow-up growth studies are accessible through the NICHD Data and Specimen Hub (N-DASH) https://dash.nichd.nih.gov/study/228833.
