# Supplementary material for: Zinc Supplementation Initiated Prior to or During Pregnancy Modestly Impacted Maternal Status and High Prevalence of Hypozincemia in Pregnancy and Lactation: The Women First Preconception Maternal Nutrition Trial
Source: J Nutr. 2024 Apr 16;154(6):1917–26. doi: 10.1016/j.tjnut.2024.04.018 (PMC11217030; doi:10.1016/j.tjnut.2024.04.018)
Supplement: Multimedia component 1 [file mmc1.pdf]

Zinc status and high prevalence of hypozincemia are inconsistently improved by supplementation during pregnancy and early lactation: the Women First Preconception Maternal Nutrition Trial
Kemp, et al

Supplemental Figure 1. Consort diagram of Women First participants in Guatemala, India and Pakistan

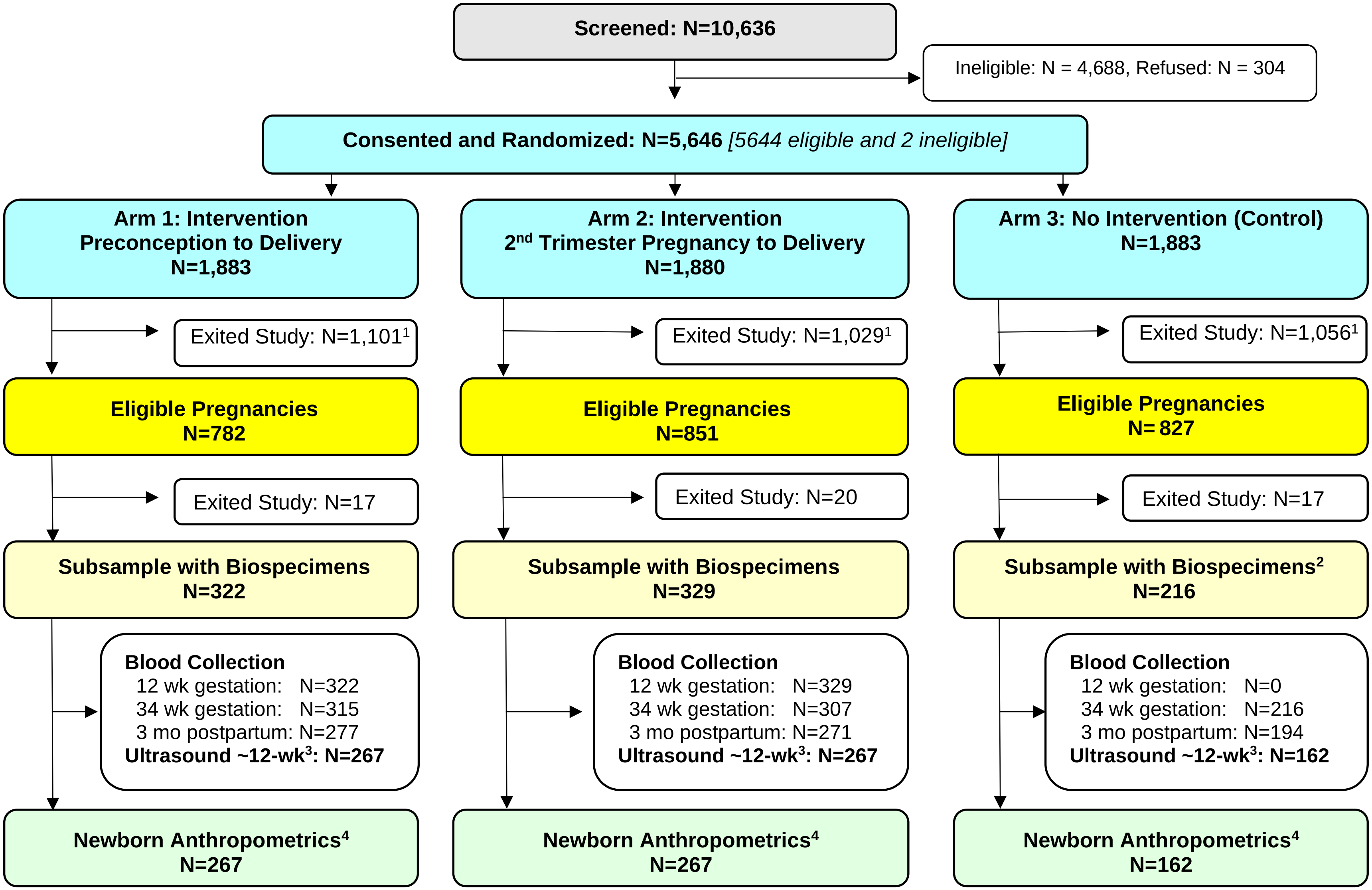

Zinc status and high prevalence of hypozincemia are inconsistently improved by supplementation during pregnancy and early lactation: the Women First Preconception Maternal Nutrition Trial
Kemp, et al

[1]Primary reason for exit was woman becoming pregnant at <3 months or not becoming pregnant before enrollment goals reached (15).
[2]No samples collected for Arm 3 in India.
[3]Gestational age (GA) at birth is defined as the GA determined by ultrasound conducted at ~12 weeks of gestation.
[4]Newborn anthropometry was obtained for live newborns with three replicate length, weight and head circumference measurements taken within 48 hours of delivery.
